# The complete mitochondrial genome of the doubled-lined mackerel *Grammatorcynus bilineatus* Rüppell, 1836 (Perciformes: Scombridae) from Beqa lagoon in Fiji

**DOI:** 10.1080/23802359.2020.1832936

**Published:** 2020-11-06

**Authors:** Nazia Tabassum, Teari Kaure, Kyeong Dong Park, Dae-Sung Lee, Hyun-Woo Kim

**Affiliations:** a Department of 4th Industrial Convergence Bionix Engineering, Pukyong National University, Busan, Republic of Korea; b KOICA-PNKU International Graduate Program of Fisheries Science, Graduate School of Global Fisheries, Pukyong National University, Busan, Republic of Korea; c Department of Marine Biology, Pukyong National University, Busan, Republic of Korea; d Department of Genetic Resources Research, National Marine Biodiversity Institute of Korea, Seocheon-gun, Republic of Korea

**Keywords:** Mitochondrial genome, next-generation sequencing, *Grammatorcynus bilineatus*, Scombridae, phylogenetic tree

## Abstract

The complete mitochondrial genome of the doubled-lined mackerel, *Grammatorcynus bilineatus,* was determined by the combination of high-throughput sequencing (HTS) and Sanger sequencing. The constructed mitochondrial genome of *G. bilineatus* was 16,537 bp in length, which harbors a canonical 37 genes (13 proteins, two ribosomal RNAs, and 22 tRNAs) and two non-coding regions (origin of light-strand replication (O_L_) and the D-loop control region). Among 38 genes, nine were encoded on its light strand (L), while the other 28 were on its heavy strand (H). Besides COX1 (GTG) and ATP6 (CTG), the other eleven protein-coding genes (PCGs) begin with a typical start codon (ATG). The phylogenetic tree showed that *G. bilineatus* was not clustered with the other species in the Scombridae, forming a clade for *Grammatorcynus*. The genetic information of *G.bilineatus* will provide useful information for the scientific management and conservation of the species in the genus.

According to FishBase (www.fishbase.org), *Grammatorcynus bilineatus* is one of two species in the genus *Grammatorcynus*, which is widely distributed in the Indo-Pacific from the Red Sea to the Andaman Sea (Collette and Gillis 1992). The higher gill raker numbers (18–24) and larger eye size of *G. bilineatus* have been known as the main morphological characters, which are distinct from its sole relative species, *Grammatorcynus bicarinatus* (Silas 1963). However, the habitat of both species shares in Fiji, and their genetic information are essential to understand the sustainable stock size and long-term fishery effect on this species (McPherson 1997). We here report the complete mitochondrial genome of *G. bilineatus,* which was collected from the coastal water of Fiji.

The specimen was collected from the coastal water of Beqa in Fiji (18°22'38"S, 177°58'25"E). The dissected muscle was stored in the 99% ethanol at −20 °C before the purification of the genomic DNA. The identification of species was confirmed by 99.53% identity to *G.bilineatus* (KF009597) in COI sequence. The extracted DNA and muscle tissue are currently stored at the Marine Biodiversity Institute of Korea (MABIK GR00004009). For the high-throughput sequencing (HTS), the mitochondrial DNA was isolated using the kit for DNA isolation (Abcam, UK) and fragmented by Covaris M220 Focused-Ultrasonicator (Covaris Inc., San Diego, USA). A library was constructed with TruSeq® RNA library kit (Illumina, San Diego, CA), which was further analyzed by the MiSeq platform (Illumina, San Diego, CA). Geneious software 11.0.2 (Kearse et al. 2012) was applied to assemble the entire mitochondrial genome of *G.bilineatus*. tRNAScan-SE software was used to obtain the secondary structures of 22 tRNAs (Lowe and Chan 2016). The software program MEGA7.0 was used to construct a phylogenetic tree with minimal evolution algorithm and 1000 bootstrap replicates (Kumar et al. 2016).

The complete mitochondrial genome of *G.bilineatus* (16,537 bp, MT680627) contained 37 genes, including 13 protein-coding genes (PCGs), two ribosomal RNAs (12S rRNA and 16S rRNA), and 22 tRNAs. Two non-coding regions, the origin of light-strand replication (O_L_) and the putative control region, were also observed. One PCG (ND6) and eight tRNAs were located on the light (L) strand, while the other 28 genes were encoded on the heavy (H) strand. The ratio of A + T to C + G was 53.2% and 46.8%. Besides COX1 (GTG) and ATP6 (CTG), the other eleven PCGs begin with a typical start codon (ATG). Incomplete stop codons were identified in six genes, including ND2 and COX3 (TA–) and COX2, ND3, ND4, and Cytb (T––). All tRNA genes displayed a clover-leaf pattern except for tRNA^Ser-GCT^, which lacked a dihydrouridine arm.

The phylogenetic analysis revealed that *G. bilineatus* did not cluster with others in the family Scombridae, forming a distinct genus clade (Figure 1). Among the compared species in the family, *G. bilineatus* showed 84.40% and 83.79% identity to *Katsuwornus pelamis* and *Auxis thazard,* respectively. The genetic information of *G.bilineatus* will provide useful information for the scientific management and conservation of the species in the genus.

**Figure 1.**
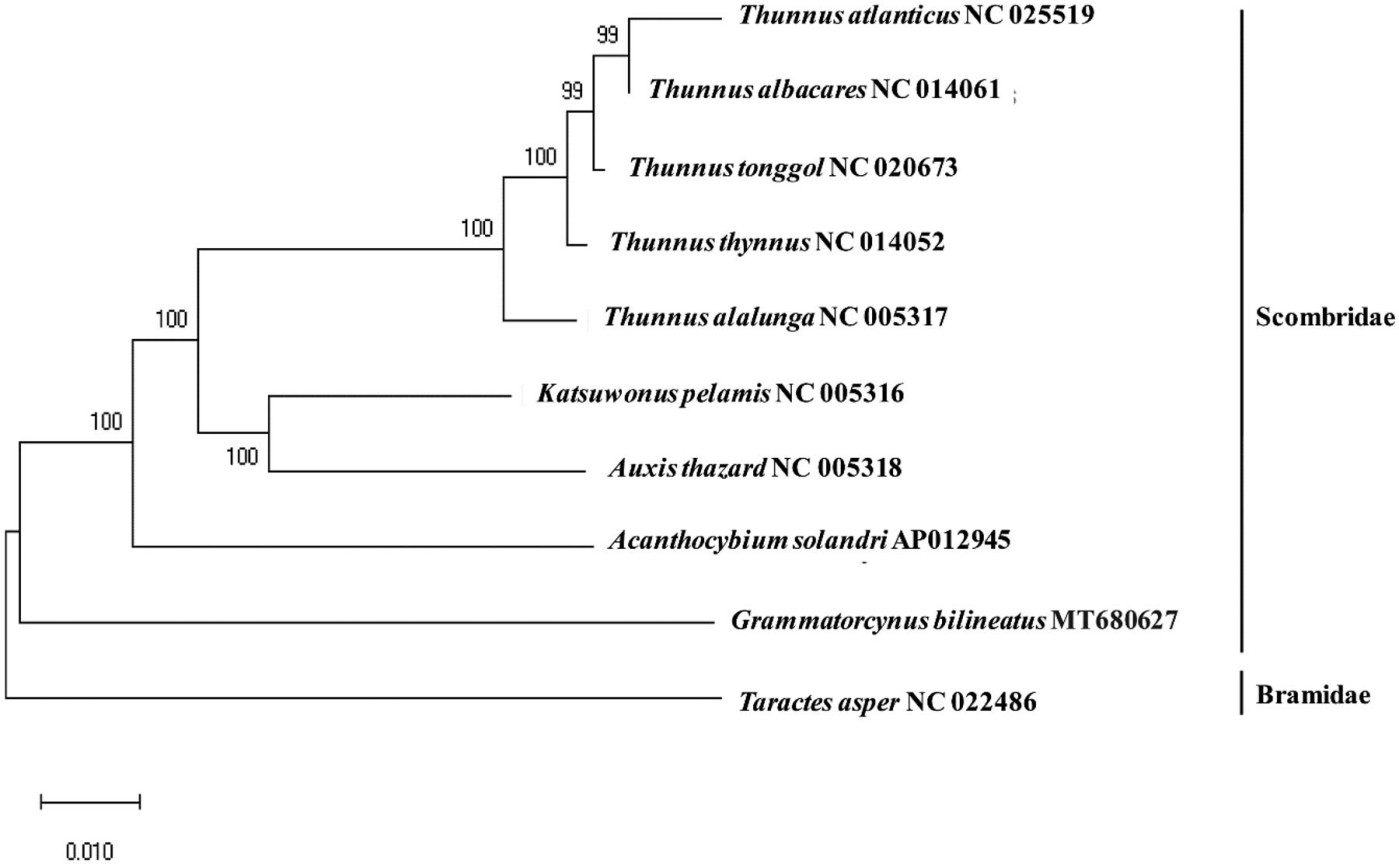
Phylogenetic relationship of *Grammatorcynus bilineatus* with fish in the family Scombridae: A phylogenetic tree with the complete mitochondrial genome in the family Scombridae with Minimum Evolution (ME) algorithm (1000 bootstrap replicates). *Taractes asper* was used as an outgroup member.

## Data Availability

The data that support the findings of this study are available in [GenBank database] [*Grammatorcynus bilineatus*] at [https://www.ncbi.nlm.nih.gov/nucleotide/] with a reference number [GenBank Number: MT680627].
